# Pathways from health beliefs to treatment utilization for severe depression

**DOI:** 10.1002/brb3.1873

**Published:** 2020-10-07

**Authors:** Flavius R. W. Lilly, Hyun‐Jin Jun, Patty Alvarez, Jenny Owens, Lauren Malloy, Meghan Bruce‐Bojo, Carol Vidal

**Affiliations:** ^1^ Department of Health Sciences Graduate School University of Maryland, Baltimore Baltimore MD USA; ^2^ School of Social Work University of Maryland, Baltimore Baltimore MD USA; ^3^ Division of Student Affairs University of Maryland, Baltimore Baltimore MD USA; ^4^ Department of Health and Social Innovation Graduate School University of Maryland, Baltimore Baltimore MD USA; ^5^ Department of Development and Alumni Relations School of Medicine Johns Hopkins University Baltimore MD USA; ^6^ Department of Psychiatry and Behavioral Sciences School of Medicine Johns Hopkins University Baltimore MD USA

**Keywords:** depression, epidemiology, treatment

## Abstract

**Background:**

Untreated depression is associated with negative behavioral, psychosocial, and physical outcomes leading to socioeconomic costs, disability, and premature mortality. Research has not yet fully developed intervention models to increase the utilization of mental health treatments. The objective of the current study was to characterize the pathways linking health beliefs to treatment utilization among depressed young adults.

**Methods:**

Data were collected in 2017 from 53,760 college students at 54 universities in the United States. Among the respondents, 5,343 screened positive for moderately severe to severe depression. Becker's Health Belief Model (HBM) was the guiding theoretical paradigm. Confirmatory factor analysis and structural equation modeling (*SEM*) were conducted to elucidate treatment‐seeking behavior based on health beliefs (perceived severity, perceived benefit, perceived barriers, self‐efficacy, and cues‐to‐action) while controlling for relevant sociodemographic covariates.

**Results:**

Depression treatment utilization was significantly associated with all domains of the HBM. *SEM* parameter estimates indicated that higher levels of perceived severity, self‐efficacy, and cues‐to‐action were associated with greater depression treatment utilization, whereas perceived benefits and perceived barriers were associated with lower depression treatment utilization.

**Conclusions:**

The HBM may be useful to predict the frequency of seeking treatment by individuals for depression. However, individualized intervention strategies targeting different aspects of the HBM are needed to promote help‐seeking behaviors in young adults with depression.

## Graphical abstract

The objective of this study was to characterize the pathways linking health beliefs to treatment utilization among 5,343 young adults who screened positive for severe depression. Improved depression treatment utilization was significantly associated with higher levels of perceived severity, self‐efficacy, and cues‐to‐action, whereas perceived benefits and perceived barriers were associated with lower depression treatment utilization. Health beliefs are useful to predict the frequency of seeking treatment by individuals for depression.

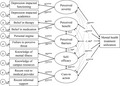

## 1 INTRODUCTION

Depression among young adults has been recognized as a serious public health concern in the United States (US) because of high prevalence rates (24%–48%) and the negative impact related to co‐occurring health and behavioral issues such as substance use (Walters et al., 2018), anxiety (Kraft et al., 2019), premature mortality (Knox et al., 2000), and suicide (Corrigan et al., 2014). Individuals with depression are more socially withdrawn and engage less frequently with others to resolve conflict (Walters et al., 2018), while also experiencing significant life‐altering events such as changes in educational status, work, and romantic relationships in their transition to adulthood (Ibrahim et al., 2013). Even further, students with depression are also at higher risk of having a lower GPA and withdrawing from college prior to graduation (Ibrahim et al., 2013).

Despite an increasing prevalence of depression among young adults (Twenge et al., 2019) and expected positive outcomes from treatments (Corrigan et al., 2014), nearly half of college students with depression do not use mental health services (Lipson et al., 2018). Unfortunately, the longer an individual remains untreated, the more likely it is that they will have negative behavioral, psychosocial, and physical outcomes. These outcomes may result in substantial socioeconomic costs (Kessler, 2012) and disability, which can also prevent the achievement of age‐ and culture‐appropriate goals (Corrigan et al., 2014). Existing research in this area has not yet fully investigated solutions that could break this cycle and lead to better utilization of mental health treatments. The high prevalence of depression coupled with insufficient treatment galvanizes the need to better understand the factors that contribute to mental health treatment utilization. One possible avenue would be to investigate the health beliefs of depressed young adults and what might influence their treatment‐seeking behaviors.

Accordingly, we address a question of critical importance: what are the specific pathways by which health beliefs influence treatment‐seeking behavior among depressed young adults? In this study, we used Becker’s (1974) Health Belief Model (HBM) to specify pathways by which health beliefs influence treatment‐seeking behavior among depressed college students. The HBM examines the mechanisms by which individuals choose to engage in treatment‐related actions in response to symptoms of depression. The HBM describes an individual's perceptions of susceptibility and severity of a disease; perceived benefits and barriers associated with engaging in health actions; self‐efficacy to take or maintain health‐related behaviors (Janz & Becker, 1984); and cues‐to‐action that trigger actions to alleviate the disease.

## 2 MATERIALS AND METHODS

### 2.1 Procedures and participants

In 2017, an observational cross‐sectional survey was administered to students (*N* = 53,760) at 54 participating universities in the US via email. Students with moderately severe to severe depression (*n* = 5,343) assessed by the Patient Health Questionnaire‐9 (PHQ‐9) (Spitzer et al., 1999) were extracted for analysis to determine health beliefs that contributed to their utilization of depression treatment. The PHQ‐9 assesses symptoms experienced in the past two weeks using the diagnostic criteria for a major depressive episode in the *Diagnostic and Statistical Manual of Mental Disorders, Fourth Edition (DSM–IV)* and has been validated as being highly correlated with depression diagnosis by mental health professionals in a variety of populations (Henkel et al., 2004; Kroenke et al., 2001). The maximum score of the PHQ‐9 is 27 with higher scores corresponding to more severe depression. Scores of 5, 10, 15, and 20 represent cutoff points for “mild,” “moderate,” “moderately severe,” and “severe” depression, respectively. When PHQ‐9 scores are greater than or equal to 15, active treatment with pharmacotherapy and/or psychotherapy is indicated (Kroenke & Spitzer, 2002). The sample was limited to depressed students who should be in treatment (i.e., those with scores ≥15) since the focus of this study was on the health beliefs which lead to active treatment utilization. The study was approved by each participating university's Institutional Review Board and each participant gave informed consent in accordance with the Declaration of Helsinki.

### 2.2 Measures

*Mental health treatment utilization* was assessed as a binary outcome using respondents’ self‐report of receiving any mental health counseling or therapy from a health professional and taking any psychotropic prescription medications in the past year. Those who received any therapy or medication in the prior 12 months were considered mental health treatment users.

Multiple items were used to characterize various domains of the HBM. These domains were derived from a principal component analysis using Varimax rotation with Kaiser normalization yielding five distinctive constructs, representing the HBM latent variables: (a) *perceived severity of depression* indicating beliefs in the extent that depression hurts their everyday and academic functioning; (b) *perceived benefits* of seeking treatment for depression indicating one's beliefs in the effectiveness of psychotherapy and medications; (c) *perceived barriers* to seeking treatment for depression comprising beliefs about the stigma associated with seeking care and the failure to perceive the threat of their depression; (d) beliefs in one's ability, or *self‐efficacy* to seek treatment, consisting of knowledge about mental illness and about accessibility of campus mental health resources; and (e) *cues‐to‐action* such as a recent visit with a medical provider (e.g., a primary care doctor) and the extent of nonclinical supports for emotional health.

The construct of *perceived severity* included two variables: *Impact of depression on functioning* assessed the level of difficulty in working, taking care of things at home, or getting along with others due to depression (1 = not difficult at all to 4 = extremely difficult) and dichotomized (0 = not difficult, 1 = difficult); *Mental health impact on academic performance* was measured by asking the frequency of feeling that mental difficulties hurt their academic performance in the past month (0 = no days, 1 = 1–2 days, 2 = 3–5 days, 3 = 6 or more days), coded as a dichotomous variable (0 = none, 1 = more than one day).

*Perceived benefits* were assessed using two items regarding *belief in treatment effectiveness related to counseling* (How helpful on average do you think therapy or counseling is, when provided competently, for people your age who are clinically depressed?) and in *medication* (How helpful on average do you think medication is, when provided competently for people your age who are clinically depressed?) with a 4‐point Likert scale from (1 = not at all helpful to 4 = very helpful) and dichotomized (0 = not helpful, 1 = helpful).

*Perceived barriers* were assessed by *perceived mental health treatment stigma* and by *failure to perceive the significant threat* of severe depression. *Personal stigma* toward people who received mental health treatment was measured by a single item adapted from the Discrimination‐Devaluation Scale (DDS) (Link et al., 1989). Respondents were asked to rate their level of agreement with the statement: “I would think less of a person who has received mental health treatment.” The item was rated on a 6‐point Likert scale (0 = strongly agree to 5 = strongly disagree), reverse‐coded and dichotomized (0 = disagree, 1 = agree). To assess perceived threat of depression, respondents identified whether they needed help for emotional or mental health problems in the past year, which was taken from the National Healthcare for Communities Study on mental healthcare utilization (Wells et al., 2003). The respondents who disagreed that they needed help were coded as *failing to perceive the threat* of depression, whereas those who agreed were coded as perceiving the threat. After reverse coding, a dichotomous variable was created (0 = perceived threat, 1 = failed to perceive threat).

The variable of *self‐efficacy* consisted of *knowledge of mental illness* and *knowledge of campus resources*. Respondents were asked to identify how knowledgeable they are about mental illnesses relative to the average person (1 = well above average to 5 = well below average) and whether they would know where to go on campus if they needed to seek professional help for their mental or emotional health (0 = strongly agree to 5 = strongly disagree). Both items were reverse‐coded and dichotomized.

To assess *cue‐to‐action*, respondents were asked to identify whether they had a *recent visit to a medical provider* (e.g., a primary care doctor or other type of doctor) for a checkup for any other medical reason, or received *recent informal support* (i.e., nonclinical counseling or support) for mental or emotional health in the past year. These items were dummy coded.

Sociodemographic variables included *sex* at birth (female, male, intersex), *race/ethnicity* (non‐Hispanic White, non‐Hispanic Black, non‐Hispanic Asian, Hispanic, or multiracial/other), *sexual orientation*, *age*, *importance of religion in life*, and US *citizenship* coded as dichotomous variables for data analytic purposes. Due to the small sample size, individuals who identified as intersex (*n* = 6, 0.1%) were excluded from the final data analysis.

### 2.3 Statistical analysis

Data analysis was conducted using the Statistical Package for the Social Sciences (SPSS) software version 24 for checking missing data, multiple imputation, principal component analysis, descriptive statistics, and bivariate analyses. Mplus version 7.31 was used for confirmatory factor analysis (CFA) and structural equation modeling (*SEM*). The assumption of multivariate normality was adequately met (i.e., skewness < 3; kurtosis < 10) (Kline, 2016). Prior to the main analysis, a multiple imputation procedure was used to address missing data among independent variables, which has been shown to be superior to traditional methods when data are missing due to random reasons unrelated to observed or nonobserved variables (Cox et al., 2014). Using Rubin’s (2009) guidance, five imputations were completed and pooled for all the analyses conducted in this study. We conducted descriptive analyses to describe both sample characteristics and the variables of interest. Additionally, we used bivariate chi‐square tests to assess the relationships between independent variables and mental health treatment utilization.

Structural equation modeling was conducted to evaluate the fit of measurement and the structural components of the hypothesized model regarding health beliefs and treatment utilization among depressed young adults. To examine the factor structure of the health belief domains, measurement models were first evaluated via five‐factor CFA allowing for correlations among the latent variables and subsequently incorporated into the *SEM* (Kline, 2016) with weighted least squares mean variance (WLSMV) estimator. The WLSMV estimator is appropriate to use with categorical and ordinal variables and produces robust standard errors (Byrne, 2013; Kline, 2016). The standardized factor loadings (STDY) are interpreted with binary variables (Byrne, 2013). Multiple‐fit indices were adopted to assess how well the model fit the data based on the recommendation of Kline (2016) and Hu and Bentler (1999): chi‐square (*χ* ^2^) goodness‐of‐fit index, the Comparative Fit Index (CFI) and the Tucker–Lewis index (TLI) ≥0.95; the Root Mean Square Error of Approximation (RMSEA) ≤0.06. WRMR is appropriate to use with categorical variables; a cutoff value of <1.0 is considered an adequate model fit (Hancock & Mueller, 2013). Although Kline (2015) suggests interpreting a good fit to be a nonsignificant *χ* ^2^ at a 0.05 threshold, a significant *χ* ^2^ value can be sensitive to discrepancies in model fit especially in large sample sizes (Byrne, 2013; Kline, 2015).

Lastly, *SEM* was conducted to test models in which five health belief domains, as validated by CFA, were associated with mental health treatment utilization among depressed students while controlling for relevant sociodemographic covariates. We evaluated potential conceptualizations of the HBM based on previous work elucidating potential pathways from health beliefs to subsequent behaviors (Chen & Land, 1986; Hasin et al., 2018; Liska et al., 1984). We hypothesized that after students receive mental health treatment, a fundamental alteration of the causal pathway occurs in which reciprocal relationships exist between health beliefs and the action of seeking mental health treatment (Chen & Land, 1986; Liska et al., 1984). The final model was modified from the hypothesized model based on prior literature and modification indices (MIs) (Kline, 2016). Path coefficients <0.1 indicate a small effect, those around 0.3 a medium effect, and those >0.5 a large effect (Kline, 2016). Model fit was assessed using the same multiple‐fit indices described above for the CFA.

## 3 RESULTS

### 3.1 Univariate and bivariate analyses

Approximately 10% (*n* = 5,343) of the total sample (*N* = 53,760) met criteria for moderately severe to severe depression. Among the respondents with moderately severe to severe depression, 60.8% (*n* = 3,251) sought mental health treatment in the past year with 24.1% utilizing only psychotherapy, 25.4% utilizing only medication, and 50.5% using a combination of both psychotherapy and medication. The mean age was 22.8 years (*SD* = 6.1), and more than half of the sample was female (76.0%), was heterosexual (68.1%), and had US citizenship (94.2%). Most of the sample identified their race/ethnicity as White (64.3%), followed by multiracial or another race (16.1%), Asian (9.7%), Black (5.3%), and Hispanic (4.6%). Table 1 shows the bivariate relationships of mental health treatment utilization by sociodemographic factors and the main study variables indicating that all measured variables in the HBM were significantly associated with mental health treatment utilization.

**Table 1:** Mental health treatment utilization by sociodemographic factors and HBM domains (*N* = 5,343)

	Untreated (*n* = 2,092)		Treated (*n* = 3,251)		*F*
	*N*	%	*n*	%	
Sociodemographics					
Sex at birth					24.40***
Female	1,516	37.3	2,547	62.7	
Male	574	45.1	700	54.9	
Race					81.32***
White	1,241	36.1	2,196	63.9	
Black	146	51.6	137	48.4	
Asian	278	53.6	241	46.4	
Hispanic	109	44.3	137	55.7	
Multiracial or other	318	37.1	540	62.9	
Sexual orientation					64.87***
Heterosexual	1,558	42.8	2,079	57.2	
LGBTQ	534	31.3	1,172	68.7	
Age					4.57*
18–22	1,470	40.1	2,194	59.9	
23+	622	37.0	1,057	63.0	
Importance of Religion					16.84***
Important	702	43.3	919	56.7	
Unimportant or neutral	1,390	37.3	2,332	62.7	
Citizenship					23.61***
U.S. citizen	1,931	38.4	3,104	61.6	
Non‐U.S. citizen	161	52.3	147	47.7	
Perceived severity					
Depression impacted functioning					27.10***
Difficult	2,018	38.6	3,206	61.4	
Not difficult	74	62.2	45	37.8	
Depression hurt academics					19.50***
More than one day	2,031	38.7	3,211	61.3	
No	61	60.4	40	39.6	
Perceived benefits					
Belief in therapy effectiveness					94.97***
Helpful	1,497	44.1	1,899	55.9	
Not helpful	595	30.6	1,352	69.4	
Belief in medication effectiveness					143.68***
Helpful	1,863	43.0	2,467	57.0	
Not helpful	229	22.6	784	77.4	
Perceived barriers					
Personal stigma					12.40***
Agree	149	48.7	157	51.3	
Disagree	1,943	38.6	3,094	61.4	
Perceived threat of depression					233.32***
Perceived threat	1,710	35.7	3,081	64.3	
Failed to perceive threat	382	69.2	170	30.8	
Self‐efficacy					
Knowledge of mental illness					78.16***
Average or above	1,848	37.5	3,086	62.5	
Below average	244	59.7	165	40.3	
Knowledge of campus resources					93.44***
Average or above	1,267	34.8	2,379	65.2	
Below average	825	48.6	872	51.4	
Cues‐to‐action					
Recent visit to medical provider					194.58***
Yes	1,419	34.2	2,734	65.8	
No	673	56.6	517	43.4	
Recent informal support					198.98***
Yes	1,359	33.8	2,666	66.2	
No	733	55.6	585	44.4	

* *p* < .05,

** *p* < .01,

*** *p* < .001.

### 3.2 Evaluation of the structural equation model

A preliminary evaluation of the five‐factor CFA model of HBM latent variables yielded a good fit (*χ* ^2^(24) = 53.91, *p* = .000; CFI = 0.99, TLI = 0.98, RMSEA = 0.02 [90% CI = 0.01–0.02, *p* = 1.00], WRMR = 0.90). All the factor loadings of each observed variable to underlying latent variables were statistically significant, with coefficients ranging from 0.32 to 0.86, with factor loadings in the anticipated direction, as shown in Table 2. Thus, the hypothesized measurement model appears reasonable.

**Table 2:** Parameter estimates of mental health treatment utilization

Parameter estimate	Unst.	St.	*p*
Measurement model			
Regression parameters			
Perceived severity → Depression impacted functioning	1.00	0.82	.00
Perceived severity → Depression hurt academics	0.85	0.69	.00
Perceived benefit → Belief in medication	0.81	0.70	.00
Perceived benefit → Belief in therapy	1.00	0.86	.00
Perceived barriers → Personal stigma	0.84	0.32	.00
Perceived barriers → Failure to perceive threat	1.00	0.38	.00
Self‐efficacy → Knowledge of mental illness	1.00	0.50	.00
Self‐efficacy → Knowledge of campus resources	0.66	0.33	.00
Cues‐to‐action → Recent visit to medical provider	1.00	0.42	.00
Cues‐to‐action → Recent informal support	1.51	0.63	.00
Covariance parameters			
Perceived severity ↔ Perceived barriers	−0.46	−1.48	.00
Perceived severity ↔ Perceived benefit	−0.05	−0.08	.20
Perceived severity ↔ Self‐efficacy	0.14	0.34	.00
Perceived severity ↔ Cues‐to‐action	0.22	0.64	.00
Perceived benefit ↔ Perceived barriers	0.20	0.60	.00
Perceived benefit ↔ Self‐efficacy	−0.19	−0.44	.00
Perceived benefit ↔ Cues‐to‐action	−0.13	−0.36	.00
Perceived barriers ↔ Self‐efficacy	−0.20	−1.06	.00
Perceived barriers ↔ Cues‐to‐action	−0.21	−1.31	.00
Self‐efficacy ↔ Cues‐to‐action	0.20	0.94	.00
Structural model			
Perceived severity ↔ Perceived barriers	−0.44	−2.01	.01
Perceived severity ↔ Perceived benefit	−0.02	−0.03	.62
Perceived severity ↔ Self‐efficacy	0.11	0.18	.04
Perceived severity ↔ Cues‐to‐action	0.21	0.60	.00
Perceived severity ↔ Mental health treatment utilization	0.23	0.28	.00
Perceived benefit ↔ Perceived barriers	0.16	0.71	.02
Perceived benefit ↔ Self‐efficacy	−0.17	−0.28	.00
Perceived benefit ↔ Cues‐to‐action	−0.12	−0.33	.00
Perceived benefit ↔ Mental health treatment utilization	−0.36	−0.42	.00
Perceived barriers ↔ Self‐efficacy	−0.31	−1.59	.02
Perceived barriers ↔ Cues‐to‐action	−0.14	−1.18	.01
Perceived barriers ↔ Mental health treatment utilization	−0.39	−1.46	.01
Self‐efficacy ↔ Cues‐to‐action	0.18	0.59	.00
Self‐efficacy ↔ Mental health treatment utilization	0.69	0.95	.00
Cues‐to‐action ↔ Mental health treatment utilization	0.27	0.62	.00

Note

A *p*‐value of .00 is used to represent *p* < .005. *χ* ^2^(63) = 123.24, *p* < .001, Comparative fit index (CFI) = 0.98; Tucker–Lewis index (TLI) = 0.96; Root mean square error of approximation (RMSEA) = 0.01 (90% Confidence Interval [CI] = 0.01–0.02, *p* = 1.00); WRMR = 0.81

Abbreviations: St., STDY standardized; Unst., unstandardized.

**Figure 1:**
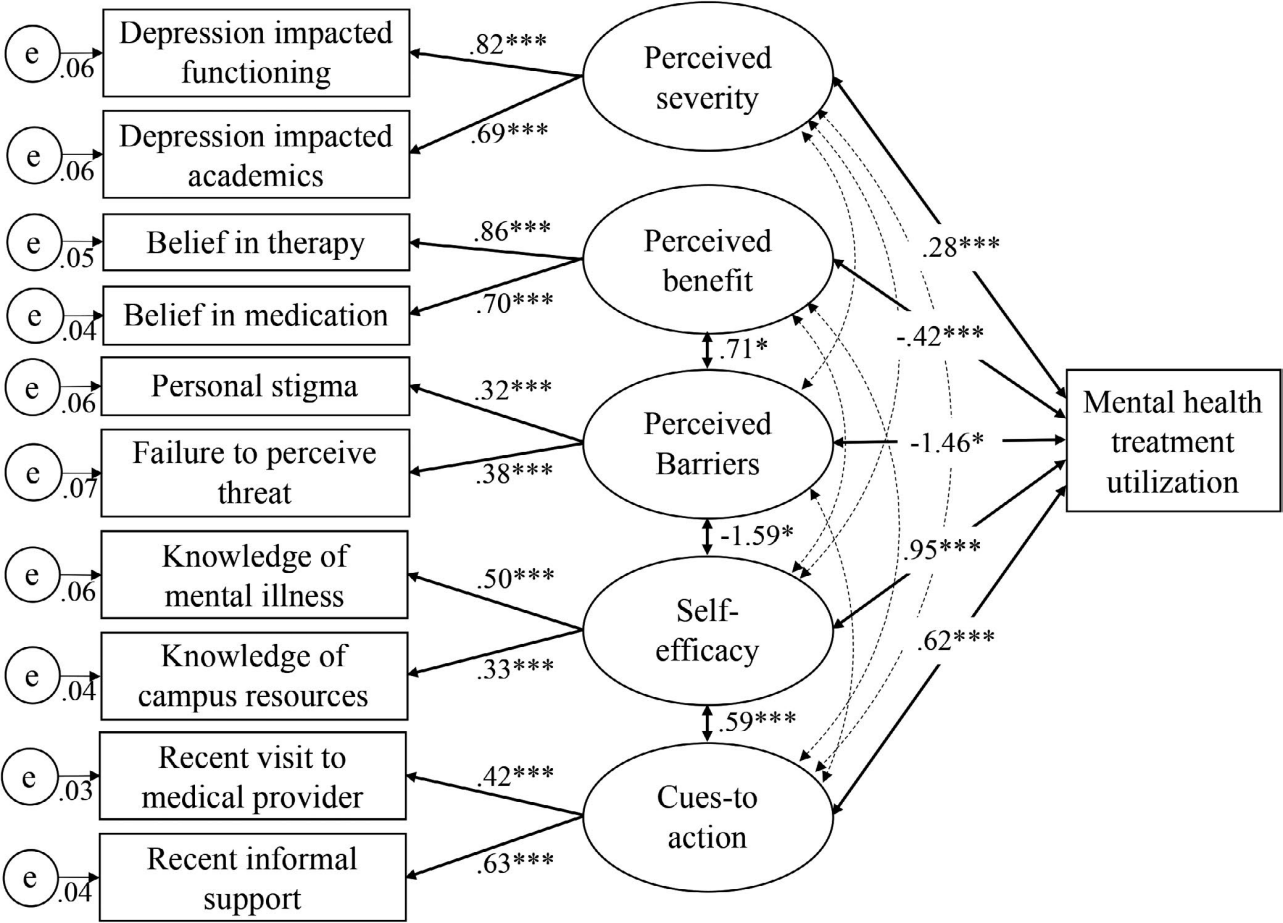
Structural equation model of the mental health utilization. Ovals present the latent variables and rectangles present the observed variables. A dashed line with arrows indicates correlations between latent variables, and those correlation coefficients were not shown in this figure but in Table 2 for display purposes. Only significant coefficients are presented. The standardized coefficients are presented. e = errors. *p < .05, **p < .01, and ***p < .001

Structural equation modeling was conducted to identify the relationships between the domains of health beliefs and mental health treatment utilization. The hypothesized model yielded a good fit to the data (*χ* ^2^(63) = 123.24, *p* = .000; CFI = 0.98, TLI = 0.96, RMSEA = 0.01 [90% CI = 0.01–0.02, *p* = 1.00], WRMR = 0.81). As shown in Table 2 and Figure 1, mental health treatment utilization was significantly associated with all domains of the HBM. Perceived severity (*r* = .28, *p* = .000), self‐efficacy (*r* = .95, *p* = .000), and cues‐to‐action (*r* = .62, *p* = .000) had statistically significant positive associations with treatment utilization, indicating that higher levels of perceived severity, self‐efficacy, and cues‐to‐action were associated with greater treatment utilization. However, perceived benefits (*r* = −.42, *p* = .000) and barriers (*r* = −1.46, *p* = .013) had statistically significant negative associations with mental health service utilization, indicating that higher levels of perceived benefits and barriers were associated with a lower level of treatment utilization. Table 3 presents coefficients of the covariates regarding the domains of health beliefs and mental health treatment utilization.

**Table 3:** Coefficients of the covariates regarding the domains of health belief and mental health treatment utilization

Variable	Perceived severity			Perceived Benefits			Perceived Barriers		
	Unst.	St.	*p*	Unst.	St.	*p*	Unst.	St.	*p*
Age	−0.19	−0.22	.01	−0.16	−0.18	.00	0.03	0.07	.53
Male	−0.49	−0.57	.00	0.26	0.30	.00	0.40	1.02	.00
Black^a^	−0.44	−0.51	.00	0.15	0.17	.05	0.40	1.02	.00
Asian^a^	−0.22	−0.26	.05	0.31	0.35	.00	0.40	1.03	.00
Hispanic^a^	−0.27	−0.32	.08	0.06	0.06	.49	0.02	0.04	.88
Multiracial/Other^a^	−0.22	−0.26	.02	0.25	0.29	.00	0.07	0.19	.22
LGBTQ	0.07	0.08	.40	−0.12	−0.14	.00	−0.32	−0.82	.00
Importance of religion	0.01	0.01	.90	−0.03	−0.04	.36	0.08	0.20	.09
Non‐U.S. citizen	−0.25	−0.29	.05	0.15	0.18	.05	−0.05	−0.13	.62

Variable	Self‐efficacy			Cues‐to‐Action			MH Tx Utilization		
	Unst.	St.	*p*	Unst.	St.	*p*	Unst.	St.	*p*
Age	0.10	0.13	.10	−0.05	−0.10	.08	0.14	0.13	.00
Male	−0.29	−0.37	.00	−0.39	−0.78	.00	−0.20	−0.20	.00
Black^a^	−0.35	−0.45	.00	−0.35	−0.71	.00	−0.38	−0.37	.00
Asian^a^	−0.10	−0.12	.26	−0.34	−0.69	.00	−0.38	−0.37	.00
Hispanic^a^	−0.54	−0.70	.00	−0.33	−0.66	.00	−0.20	−0.19	.02
Multiracial/Other^a^	0.01	0.01	.93	−0.01	−0.01	.85	−0.02	−0.02	.65
LGBTQ	0.22	0.29	.00	0.11	0.21	.00	0.27	0.26	.00
Importance of religion	−0.13	−0.17	.02	−0.02	−0.04	.50	−0.09	−0.09	.02
Non‐U.S. citizen	−0.54	−0.70	.00	−0.26	−0.52	.00	−0.17	−0.16	.04

Note

A *p*‐value of .00 is used to represent *p* < .005.

Abbreviations: MH Tx, Mental health treatment utilization; St., STDY standardized; Unst., unstandardized.

^a^ Reference = non‐Hispanic White.

## 4 DISCUSSION

Depression is a serious public health problem. As a result, there is an urgent need for research providing a greater examination of pathways to treatment utilization (Corrigan et al., 2014). Using the HBM, this study examined the association between health beliefs and mental health treatment utilization. Our findings corroborate and provide evidence for the guiding theoretical framework showing that certain health beliefs and behaviors may enhance treatment utilization among depressed young adults. In support of the theoretical model, greater perceived severity of depression, greater self‐efficacy, more cues‐to‐action, and less perceived barriers led to more treatment utilization. However, higher treatment utilization was associated with lower levels of perceived benefits. Furthermore, self‐efficacy and perceived barriers had the strongest associations with treatment outcomes.

In this sample, 10% of respondents met the criteria for moderately severe to severe depression, consistent with a recent US national survey (Hasin et al., 2018) that found a 10.4% prevalence of major depressive disorder (MDD) in the population. A majority of the young adults in our sample (61%) sought treatment, which is slightly less than the 69% of US adults who report some instance of treatment over their lifetime for MDD (Hasin et al., 2018). This small difference is likely due to the relatively young age of our sample, and to the fact that we accounted only for treatment in the past year. Although the recommended treatment for moderately severe to severe depression is a combination of therapy and medication, only half of our sample received both. Previous research (Ennis et al., 2019; Lipson et al., 2018) reported that being a woman was associated with higher levels of treatment utilization. We also found that being older, White, LGBTQ, nonreligious, and a US citizen were all associated with higher levels of treatment utilization.

Symptom severity and impairment are well‐known factors associated with seeking treatment for mental health problems (Jones et al., 2015), which was confirmed in the current study. Perceived barriers (*r* = −1.46) had the highest coefficient among all the structural coefficients, indicating its importance in the associative process between health beliefs and depression treatment. A significant body of research has examined stigma and how it affects an individual's desire to seek mental health treatment. While early recognition of mental health disorders might lead to better treatment, individuals with mental illness are often stigmatized (Corrigan et al., 2014). In a sample cross‐national sample of first‐year college students, Ebert et al. (2019) found attitudinal barriers to be more common than structural barriers when considering access to mental health services. Factors like embarrassment or wanting to handle mental health problems on one's own decreased the odds of intention to seek treatment. The current study extends the findings of Ebert et al. (2019) by examining barriers‐to‐care as well as every other construct of the HBM as predictive pathways to treatment. All the elements of the model and their interactions are key to understanding treatment utilization among the severely depressed. Relatedly, all the HBM constructs were considered collectively, controlling for each using structural equation modeling (*SEM*), which is ideal when testing theories that include latent variables allowing for calculations of strength, direction, and significance of relationships.

Previous research has shown inconsistencies in the effects of self‐efficacy on treatment‐seeking behaviors, with some studies showing that lower versus higher self‐efficacy was associated with seeking treatment (Jackson et al., 2007; Keeling et al., 2020), and others finding no association (Andersson et al., 2014). More investigation is needed of the relationship between self‐efficacy and differences in treatment‐seeking behaviors by race and sex. Our findings indicate that future research should consider the large number of students who did not seek treatment, as well as those who are male and in racially/ethnically diverse groups such as Asian American and Black American students.

Perceived benefits were unexpectedly associated with less treatment utilization. Individual's treatment expectations and prior negative experiences with mental health services may have led to subsequent disengagement from treatment. This finding differed from the assumption of the HBM that health beliefs precede and activate health behaviors like treatment‐seeking; in fact, the present study revealed a bidirectional relationship between perceived benefits and treatment utilization. The action of receiving treatment for depression may decrease individuals’ confidence in the effectiveness of therapy and medication. Individuals who have not experienced treatment may have higher expectations for how much therapy and medication can help their depressive condition. However, upon receiving treatment, the individual learns that the status of their depression is more difficult to improve than expected, which may make them perceive treatment as less effective. There is a precedent in the literature for this bidirectionality. For instance, patient expectations about treatment are a well‐known factor influencing treatment outcomes for depression (Kirsch & Sapirstein, 1998; Walsh et al., 2002). These expectations upon entering treatment are based on the patient's understanding of the treatment, their illness, and their past experiences with treatment (Rutherford et al., 2010). A qualitative study with young adults who engaged in at least four months of treatment compared their expectations of psychiatric treatment to their actual experiences. Participants expected a cold and serious atmosphere and a directive therapeutic relationship with a “quick fix.” They found treatment to be more complex than anticipated and most had negative experiences at some point in their care (Armstrong et al., 2019). Our findings suggest that there may be benefits in debriefing during and after treatments. Mental health providers could inquire about other aspects of the HBM such as if the patients feel the treatment is working, what barriers they face, and how confident they feel about their treatment.

These findings should be interpreted within the context of several limitations. First, the sample consisted of cross‐sectional data, limiting our ability to establish causal relationships among the variables. Additionally, we did not use specific measures for the health belief domains, but rather latent variables developed through factor analysis. The measures were self‐reported, which may produce recall bias or social desirability issues. Finally, there is still a lack of understanding and need for more research on the applicability of the model to racially/ethnically diverse communities. Despite these limitations, the present study uncovers important pathways through which health beliefs and behaviors may influence treatment‐seeking behavior among young adults. These findings yield valuable insights for future research on the social and psychological correlates of treatment‐seeking behavior of depressed young adults. Moreover, using structural equation modeling that accounts for measurement error, the present study parsed out potential bidirectional relationships in which the act of engaging in depression treatment may influence perceptions of susceptibility and severity, barriers to treatment, self‐efficacy, perceived benefits, and cues‐to‐action.

## 5 CONCLUSION

Given disparities in mental health treatment utilization among young adults who have depression, our findings highlight the need to develop interventions targeting different aspects of the HBM in different populations. Intervention strategies to promote help‐seeking behaviors in young adults with depression should focus on: (a) stigma reduction and education campaigns, (b) screening and linkage programs (i.e., identifying students in distress and connecting them with resources), (c) education programs to enhance self‐efficacy (e.g., increasing knowledge about mental illness and providing information about campus resources), and (d) gatekeeper training of individuals who frequently interact with students (e.g., residence life staff, faculty, advisers) (Eisenberg et al., 2012). Additionally, direct outreach should focus on racially/ethnically diverse communities.

## CONFLICT OF INTEREST

The authors declare there are no conflicts of interest.

## AUTHOR CONTRIBUTIONS

Flavius Lilly and Carol Vidal conceived of the idea for this manuscript, developed the theoretical framework, wrote the discussion section, and supervised the findings of this work. Flavius Lilly and Hyun‐Jin Jun developed the methodological approach, performed computations, and prepared the methods section of the manuscript. Patty Alvarez, Jenny Owens, Lauren Malloy, and Meghan Bruce Bojo contributed to the interpretation of the results, provided valuable input regarding the construction of the theoretical framework, and contributed early drafts of the introduction and background sections of the manuscript. All of the authors provided critical feedback and helped shape the research, analysis, and final version of the manuscript.

### Peer Review

The peer review history for this article is available at https://publons.com/publon/10.1002/brb3.1873.

## Data Availability

The data that support the findings of this study were derived from a public domain resource available in the Healthy Minds Network at https://healthymindsnetwork.org/research/data-for-researchers/
